# Prevalence, Best Practice Use, and Member Engagement on School Mental Health Teams

**DOI:** 10.3390/bs14080716

**Published:** 2024-08-16

**Authors:** Katelyn Wargel-Fisk, Amy M. Kerr, Margaret D. Hall, Nicole S. Litvitskiy, Paul D. Flaspohler, Amanda L. Meyer

**Affiliations:** Department of Psychology, Miami University, Oxford, OH 45056, USA; kerram2@miamioh.edu (A.M.K.); hallm3@miamioh.edu (M.D.H.); litvitns@miamioh.edu (N.S.L.); flaspopd@miamioh.edu (P.D.F.); meyeral6@miamioh.edu (A.L.M.)

**Keywords:** school mental health, teams, team engagement, program implementation, multi-tiered school mental health program

## Abstract

School mental health (SMH) teams have been widely recommended to support multi-tiered mental health program implementation in schools. Available research suggests emerging best practices that promote effective SMH teaming and indicates the importance of having team members who are highly engaged (e.g., actively involved, retained on the team). Despite evidence that these factors improve team functioning, there is limited knowledge of SMH team prevalence, best practice use, and factors impacting member engagement among a diverse sample of elementary schools. This study surveyed a cross-sectional sample of elementary principals (*n* = 314) across the United States whose schools implement multi-tiered SMH programs. Most principals (89%, *n* = 280) reported using teams to organize these programs. Schools in urban/suburban communities, with 300 or more students, or with specific school funding for SMH activities were more likely to have SMH teams. Only one-third of principals reported that their team members participated in related training. Other SMH team best practices were commonly reported (by two-thirds or more teams). Results of a linear regression model indicate that larger teams (six or more members) and teams with access to resources had significantly higher member engagement scores. The study’s findings provide recommendations for practice and future research directions.

## 1. Introduction

High levels of youth mental health concerns [1] emphasize the need for accessible and integrated mental health services across communities [2,3]. Prior research indicates that schools play a crucial role in providing such services to promote children’s social, behavioral, and mental well-being [4,5]. To effectively support children’s mental health, best practices recommend that schools organize multiple services among three tiers; universal prevention for all students (tier one), indicated intervention for students with known risk factors (tier two), and targeted intervention for students with identified difficulties (tier three [6]). However, few schools have appropriate resources and capacity to implement quality services along this continuum and achieve positive student outcomes [7,8]. Interdisciplinary school mental health (SMH) teams have been widely recommended as a tool that schools can use to pool resources, develop a strategic plan, and implement high-quality multi-tiered SMH services that promote student well-being while fitting the schools’ capacity (e.g., [9,10,11,12,13,14,15,16,17]). The current study surveyed a cross-sectional sample of elementary school principals across the United States (US) to learn more about SMH team prevalence, best practice usage, and factors that impact member engagement. The results aim to provide practical guidance for schools to better support students’ mental health and to inform future research directions.

The current study focuses on SMH teams that provide support for multi-tiered SMH programs in elementary schools within the United States. SMH teams often operate within one school building and vary considerably in size, composition, and task focus to meet a school’s specific needs. Among teams described in the current literature (e.g., [13,15]), team members often include school staff/faculty with expertise in mental health and/or program implementation, such as school counselors, psychologists, social workers and/or intervention specialists, and school administrators. General education teachers, community partners, and family advocates may also be team members. Team leaders may include school administrators, district personnel, a school board representative, or another member who is appointed or agreed upon by the group. SMH teams’ goals vary, but generally they aim to help a school implement school-wide mental health promotion activities (e.g., anti-bullying programming, a social–emotional learning curriculum), targeted programs for high-risk students (e.g., “lunch bunch” groups or grief groups), and individual intervention for students with identified needs (e.g., one-on-one mental health therapy). Key tasks for SMH teams include providing information to school staff and families about available mental health programming (e.g., through flyers, training, meetings, etc.), organizing resources, implementing SMH programs, and evaluating program impact for students. More detailed examples of multi-tiered SMH teams are provided in McIntosh and Goodman [13], NCSMH [15], and Splett and colleagues [17,18].

The following sections summarize the existing literature on multi-tiered SMH teams. First, successful examples of multi-tiered SMH teaming are described, followed by potential challenges and pitfalls. A summary of current evidence-based strategies for effective SMH teaming follows, including descriptions of emerging “best practices” and the impact of member engagement on team productivity. The goals of the current study aim to address current research gaps and learn more about (1) the prevalence of SMH teaming among schools who have implemented MTSS, (2) the degree to which SMH teaming best practices are adopted among schools using teams, and (3) school and team factors that impact team member engagement. Study results are presented along with implications and recommendations for researchers and school-based practitioners who support multi-tiered SMH programs.

### 1.1. Efficacy of Teaming to Support Multi-Tiered SMH Programs

Schools have consistently used teams to support student learning and well-being. Specific to teams supporting multi-tiered SMH programs, the current literature indicates that high-functioning teams can positively support schools’ SMH goals, though not all teams are “high-functioning,” which can impact their effectiveness.

Teams supporting multi-tiered SMH services have demonstrated efficacy in increasing schools’ capacity and providing positive impact to students. Related to capacity, multi-tiered SMH teams have been reported to help allocate resources across multiple priorities and reduce resource competition between classrooms, school buildings, and districts [19]. In two qualitative studies, members of multi-tiered SMH teams reported that involvement with the team helped disperse responsibilities and decrease burnout among staff [20,21]. Team members also state that they learned of available resources within their school and community by participating in multi-tiered SMH teams, thus increasing feelings of efficacy when referring students or developing classroom behavior plans [20,21,22,23]. SMH teaming has also been associated with increased multi-tiered SMH services being offered at schools [22,24] and increased SMH program sustainability [25].

Related to student outcomes, previous SMH teams report that their efforts promote positive student behaviors, enhance teachers’ abilities to address challenging behaviors, and lead to higher levels of student learning [20,26,27]. Implementations of multi-tiered SMH teams have been associated with decreased office disciplinary referrals (ODRs) and out-of-school suspensions (OSSs [28,29]). Taken together, there is emerging evidence indicating that a teaming approach can be associated with increased capacity and positive student outcomes.

Despite potential benefits, teaming can be associated with inconsistent outcomes [30]. Coordination costs (e.g., time spent in meetings, navigating disciplinary silos) can reduce teams’ positive impact [31,32] and result in schools spending more resources without providing significant benefit to students [30,33]. Ineffective teaming is associated with increased staff burnout and turnover [20,34] and can impact trust between colleagues [35]. This is a critical concern, given that the recommendation to use SMH teams stems from a need to build school capacity. To promote this goal and avoid further straining school resources, several studies have begun investigating team characteristics and practices that differentiate effective from ineffective SMH teaming, as described in the following section.

### 1.2. Effective SMH Teaming Strategies

Several studies have assessed the impact of team inputs (e.g., processes, support systems, training) on team outcomes (e.g., team functioning, student outcomes) in a multi-tiered SMH setting. A review of these articles (presented in detail as a systematized literature review in prep [36]) along with a consultation of existing conceptual guidelines for SMH teams has led to six characteristics being identified as emerging “best practices” (BPs) for effective SMH teaming. Factors identified as impacting teams’ effectiveness include (1) participation in “how-to-team” training, (2) interdisciplinary presence, (3) access to a school-wide data system, (4) administrator engagement, (5) access to relevant resources, and (6) use of evidence-based practice (EBP) guidelines. These characteristics are considered emerging BPs as initial research has highlighted their potential positive impact on SMH teaming (as described below). In addition to these six BP characteristics, the current literature identifies member engagement (e.g., retention and active participation on teams) as an additional factor that impacts team functioning and outputs (e.g., [22,25]).

The following summary of BP and team engagement for SMH teaming provides a foundation for the current study. While the teaming literature exists outside of the school setting (e.g., medicine, business), findings of these studies may not consistently generalize to school settings. Schools have unique environmental factors (e.g., funding limitations, staffing shortages, policies tied to local governance) that differ from business and medical settings. In addition, school-based mental health programs are not consistently required or supported by schools’ governing bodies (e.g., state governments, local school boards). Therefore, many schools develop SMH teams as “side projects” that compete with their mandated priorities (e.g., student learning) for time and resources. The summary below emphasizes findings from research conducted specifically with SMH teams to help ensure findings are applicable to this unique context.

#### 1.2.1. SMH Teaming Best Practices (BPs)

##### “How-to-Team” Training

Across qualitative interviews [20,21], survey comments [22], and case study descriptions [23], team members shared that their prior training did not prepare them for interdisciplinary team or SMH-specific work. Six studies provide evidence that participation in standardized training called Team-Initiated Problem Solving (TIPS) supports teams in developing meeting organization and data-based problem-solving skills. These skills have been associated with positive team functioning and fewer student disciplinary problems (ODRs and OSSs) [26,28,37]. Case study and qualitative survey findings indicate that team members perceived that using TIPS skills helped improve meeting efficiency, allowing teams to better support students [28,29,38]. Data from pre–post evaluations [37] and quasi-experimental designs [26,27,28] indicated that teams who received TIPS training showed significantly increased (with moderate to large effect sizes) meeting organization and data-based problem-solving skills compared with teams who did not receive TIPS training. Taken together, these studies indicate that participating in “how-to-team” training is an important step in helping members gain skills needed to support SMH program implementation.

##### Interdisciplinary Presence

The benefits of interdisciplinary teams have been identified widely across industries (e.g., [39,40]), and these findings are consistent within the SMH setting. Cross-sectional [41], pre–post evaluation [42], and interview data [20,21] indicated that as teams had higher levels of interdisciplinary representation, they provided more SMH services across tiers, reported higher-quality teamwork, and perceived a greater ability to address a range of student behavioral problems. Cross-sectional survey data [43] showed a positive association between the level of interdisciplinary collaboration and team members’ positive regard when completing team-related tasks. These findings emphasize the importance of having multiple disciplines represented on SMH teams.

##### School-Wide Data System

School-wide data systems (that track student academic, behavioral, and mental health outcomes) are tools that allow teams to access the information needed to make programming and resource decisions [15,17]. Team members qualitatively reported that they could better identify student needs and appropriate behavioral accommodations when using data to engage in evaluation and quality improvement [22]. Cross-sectional survey data indicated a positive association between teams’ consistent data use and the sustained implementation of school-wide behavior systems [25]. Within quantitative pre–post surveys, teams reported implementing significantly more solutions [22] and a wider use of universal screening [24] after adopting structured, data-based evaluation practices. Quasi-experimental results indicated that teams who used data within a data-based problem-solving process reported significantly fewer student disciplinary reports over time (ODRs and OSSs) compared to teams who were not using a data-based problem-solving approach [28]. In qualitative survey comments, SMH team members reported that school-wide data systems helped their decision-making processes [25,28], further illustrating the importance of teams’ access to data tools.

##### Administrator on the Team

In case studies, interviews, and survey comments, SMH team members noted that present and actively involved administrators supported team success and facilitated progress towards SMH goals [20,21,23]. Conversely, administrator turnover reportedly disrupted teams’ progress and focus [22,23]. In Raia-Hawrylak and colleagues’ [44] case study, consultation support was perceived to increase administrators’ SMH-related skills, which improved teams’ fidelity in implementing evidence-based school climate programs. Findings from these studies consistently indicate the importance of administrator engagement for SMH teams’ progress.

##### Access to Relevant Resources

Research across business industries has indicated the importance of providing work teams with the resources (e.g., physical tools, funding, personnel capacity) needed to accomplish their goals [45]. Similar findings are present within the SMH team literature. When given an initial approved budget, physical space, and time within the school calendar, team members have qualitatively reported that they are better able to develop a strategic approach to implementing multi-tiered SMH programs [20]. Lacking this access creates more coordination costs, as team members must continually seek approval for funding, program adjustments, and scheduling faculty-targeted training [46]. Teams can be more efficient when given adequate resources and knowledge of their limitations so that they can plan accordingly and guide more consistent implementation processes [17,45]. Team members have commented that having independent decision-making power over school resources (within an approved budget) allows them to create a more streamlined approach to building SMH services [20,21]. The existing literature indicates that providing teams with access to and decision-making authority over resources may contribute to more efficient teaming and program implementation.

##### Evidence-Based Practice (EBP) Guidelines

Multi-tiered SMH programming based on evidence-based practice guidelines shows more positive impact compared with programming without research backing [47,48]. Specific to SMH teaming, teams using evidence-based programming frameworks (e.g., Positive Behavior Intervention Supports [PBIS] and Trauma-Informed Care [TIC]) have had success in reaching positive student outcomes [49,50]. Similarly, evidence-based evaluation practices (such as Plan Do Study Act or PDSA cycles) have helped teams identify barriers, adjust, and expand universal mental health screening [24]. Across studies, team members have qualitatively reported that using evidence-based practice guidelines helps alleviate decision-making burdens and more accurately address student needs [20,29,42], emphasizing the importance of teams’ access to evidence-based guidelines.

#### 1.2.2. SMH Team Member Retention and Active Involvement (Engagement)

Several of the BP characteristics described above indicate the importance of selecting interdisciplinary, “engaged” team members. Member engagement and experience (or members’ availability, active involvement, reliability, and attitude towards work activities) have been widely indicated to impact team or workgroup performance in business settings [51,52]. Within the context of SMH teams, the existing literature highlights several additional team staffing considerations. Qualitative [20,21,23] and cross-sectional [25] data indicate that team members who were engaged (i.e., available, actively involved, reliable, and enthusiastic about team activities), “fit” together, and had positive camaraderie also had more effective interdisciplinary collaboration and teamwork. Several members commented that active participation and positive team member relationships help facilitate clarity about member roles and responsibilities [42,43]. Members also perceived regular meeting attendance and greater contribution from members as positively impacting the team’s atmosphere and ability to make progress towards goals [21].

In addition to active engagement, member retention was associated with team progress. Qualitatively, members noted that turnover disrupted teams’ progress and focus [22,23]. Members shared that when administrators could support team members by accommodating their job responsibilities to allow for SMH teamwork, members were more likely to attend meetings, contribute to team activities, and remain a member of the team [20,21,44]. Without this strategic planning, members reported increased difficulty attending meetings and working outside of typical work hours to keep up with team responsibilities. At times, members reported that these factors led to increased burnout and decreased buy-in with SMH teaming [20,21]. These team staffing considerations emphasize the importance of considering engagement and retention when establishing SMH teams. While the term “member engagement” can encompass a variety of member attitudes and behaviors, the current study focuses on aspects of member engagement identified in the SMH literature (i.e., active participation and member retention). Further details about the conceptualization of member engagement within this study are provided in the methods section.

### 1.3. Gaps in the Existing SMH Teaming Research

While the findings of these studies provide initial guidance for multi-tiered SMH teams, limitations exist, and they highlight topics for continued research. Several gaps emerge as initial topics to address to better understand the feasibility of SMH teaming in practice and to provide a foundation for future research. These research gaps include (1) a limited quantity of empirical studies compared with conceptual recommendations, (2) a lack of understanding of team presence, member characteristics, and BP usage among varied school environments, and (3) a lack of studies on factors that impact team member engagement, specifically in SMH contexts.

First, despite frequent recommendations for using SMH teams as a strategy for supporting and organizing multi-tiered SMH efforts (e.g., [9,10,11,12,13,14,15,16,17]), only sixteen articles have documented empirical research on the use and implementation of multi-tiered SMH teams. The lack of research may reflect the challenges of conducting research in natural school settings [53]. Alternatively, it could be that the implementation of multi-tiered SMH teams is not widespread across schools and that identifying such teams for study outside of those supported by research implementation is difficult. To the authors’ knowledge, no study has yet identified the prevalence of multi-tiered SMH teams among a large, diverse sample of schools. Understanding the level of adoption of SMH teams and whether schools’ environmental factors are associated with SMH team presence may highlight schools’ readiness for a team approach.

Second, emerging evidence indicates that school environments (e.g., student need level, school size, funding mechanisms, guiding SMH framework) impact team functioning, though the variety in environmental contexts previously explored is narrow [33,47,54]. Many existing articles feature teams with common characteristics that are unlikely to be representative of the full range of schools implementing mental health programs for students (e.g., [27,29,37]). Teams represented in existing studies have primarily had prior experience successfully implementing multi-tiered SMH programs and external support through district, research, and community partners; they also used the PBIS framework to organize their SMH programs (e.g., [28,38]). Therefore, it is not clear whether the BPs identified within these studies are feasible or regularly used among schools with different contexts.

Third, team member engagement (defined in this study as members’ active involvement and retention on the team [22,23,25]) has been associated with more positive teaming outcomes. Several studies noted the importance of accommodating members’ time to allow for team activities [20,21,44], but potential impacts of other recruitment strategies, incentives, best practice usage, and school/team characteristics on member engagement have not yet been assessed. As team member engagement is a basic building block of using teams, it is critical to better understand factors that may impact team member engagement. Learning more about the prevalence of teams, their best practice usage, and factors that impact member engagement could help to identify practical strategies for schools to engage and maintain SMH team members.

### 1.4. The Current Study

To address these research gaps, a cross-sectional study collecting quantitative data to be analyzed using descriptive statistics and multiple linear regression was completed. The study included a brief survey that was sent to a large sample of public elementary school principals who were asked about the SMH programs provided at their school and whether they use teams to support implementation of these services. If teams were used, principals were prompted to share information about their teams’ structure, best practice usage, and member engagement and the overall school environment. The survey measure used in this study is unique in that it was developed using a community-based, collaborative approach [55]. The research team consulted with community practitioners (see the methods section below) to help ensure that the survey’s language and content were relevant to the practical experiences of principals, rather than reflecting academic terms that may not directly translate to SMH teams’ day-to-day experiences in schools. The survey aimed to collect data to answer the following research questions:

RQ1: What portion of schools implementing multi-tiered SMH programs report using teams to support these programs?

RQ2: What are common characteristics of SMH teams, including how often schools with multi-tiered SMH teams report using current best practices for SMH teaming?

RQ3: What best practices, team characteristics, and school characteristics relate to SMH team member engagement (as conceptualized by principals’ perceptions of members’ active involvement and retention on SMH teams)?

Regarding RQ1, given the high frequency at which this strategy is recommended, it was hypothesized that most schools that provide multi-tiered SMH activities will use teams to organize these activities. However, as previous research on SMH frameworks has emphasized the importance of interdisciplinary collaboration (e.g., [41,42]), regarding RQ2, it was hypothesized that most teams will have an interdisciplinary presence representing three or more unique disciplines. Related to RQ3, it was hypothesized that school characteristics (e.g., funding), team characteristics (e.g., team size, interdisciplinary representation), the presence of BPs, and member recruitment/incentive strategies may impact member engagement. Specifically, recruitment/incentive strategies that provide team members with protected time, funding, and training are hypothesized to be associated with stronger member engagement, based on prior research suggesting that the strategic organization of teams supports more active member participation [20,21,44].

## 2. Materials and Methods

### 2.1. Participants

Principals (*n* = 352) of public elementary schools within the US participated in this study. Principals were randomly identified from a list of all public elementary schools in the US (see procedures below). Chi-square tests (using a cutoff of *p* < 0.01) were used to compare characteristics of schools represented by principals in the present sample with the characteristics of all public elementary schools in the US [56]. Within the current sample, the percentages of large schools (501+ students), Title I eligible schools, and schools in suburbs or towns were comparable to the percentages of these schools within the full population. Schools overrepresented in the current sample included medium-sized schools (331–500 students; 41% vs. 34%) and rural schools (34% vs. 26%). Small schools (1–300 students; 25% vs. 33%) and urban schools (21% vs. 29%) were found to be underrepresented in the present sample. Geographically, schools in US Regions 5 (midwestern states; 26% vs. 17%) and 8 (northwestern states; 9% vs. 6%) were overrepresented within the current sample, while schools from Region 6 (states in the middle south; 7% vs. 14%) were underrepresented [57]. Within the current sample, schools from the other seven regions were approximately representative of the full population. While base sizes sufficiently powered quantitative analyses (see analysis section), unequal representation as well as the relatively small size of the sample may impact generalizability, as detailed in the discussion. Most participating schools (81.4%) reported having multiple funding sources specific to SMH activities. Many schools (82.1%) also reported using multiple SMH frameworks to guide activities, as shown in Table 1.

### 2.2. Measures

Data for this study were collected via a single, brief survey (content described below). In order to promote the survey’s relevance to elementary school principals’ experiences, a community-based approach was used during survey development. A group of practitioners (including two elementary school principals, a school counselor, a school intervention specialist, and a faculty member from a university’s Department of Teaching, Curriculum, and Educational Inquiry) was consulted throughout measure development. The survey prioritized brevity to reduce the burden on principals and encourage survey completion. Principals were first asked (a) whether their school provides multi-tiered (prevention and treatment) SMH services and (b) whether their school uses teams to support such services. Examples of multi-tiered SMH services were listed to help principals accurately answer these initial questions, as advised by the school-based practitioner group. Principals with SMH teams were then asked questions about their teams related to variables of interest for the study, as described below.

#### 2.2.1. Team Characteristics and Best Practices (BPs)

Multiple choice and “select all that apply” questions asked principals about their team’s size, member disciplines, partners, and whether or not they use any of the six SMH teaming BPs (interdisciplinary presence, administrator present on team, using EBP guidelines, access to school-wide data system, access to relevant resources, and participation in “how-to-team” training).

#### 2.2.2. Team Member Engagement, Recruitment, and Accommodations

This study was designed to use principals’ perceptions to better understand SMH team functioning across a large sample of schools rather than provide a deep and nuanced understanding of all individual team members’ levels of engagement on their specific school teams. To accomplish this goal, two questions assessed principals’ ease of keeping team members “engaged” in team activities and ease of keeping teams fully staffed. These questions were chosen based on the previous literature stating that member engagement and turnover impact team outcomes. In addition, the school-based practitioner team that helped develop the survey identified these as key factors in overall member engagement and team functioning. The school-based practitioner team recommended two or fewer questions be used to assess engagement in order to keep the survey brief and realistic for principals to complete. This aligned with the research teams’ goals of collecting a brief assessment of principals’ perceptions of engagement and promoting survey completion by maintaining a brief survey. Principals rated each statement on a 5-point Likert scale with higher scores indicating easier member engagement/retention. Within this sample, these items were moderately correlated (*r* = 0.568, *p* < 0.001) and had acceptable reliability (Cronbach’s α = 0.722). The engagement and retention item scores were averaged together to develop an overall member engagement score.

To measure additional staffing factors, two separate “select all that apply” questions prompted principals to report their methods for recruiting (e.g., requiring participation, seeking volunteers) and providing accommodations (e.g., bonuses, protected time) to SMH team members. Within these questions, principals could type in “other” recruiting/accommodation methods used.

#### 2.2.3. School Characteristics

Principals were asked multiple choice questions about their state, community type (rural, town, urban, suburban), school size (small 1–300, medium 301–500, large 501+), Title I status, SMH funding source(s), and SMH framework(s) used. The school size groupings are adopted from standard practices within NCES. Schools’ states were mapped onto FEMA’s ten educational regions [57]. Principals had the opportunity to select all that apply and/or to write-in “other” SMH funding and frameworks used if applicable.

### 2.3. Procedures

#### 2.3.1. Developing a Sample

A university Institutional Review Board approved this study prior to data collection. This project sought input from a nationally representative sample of principals at public elementary schools within the US. To identify a sample, public elementary schools were identified via NCES’s Common Core of Data 2020–2021 directory (the most recent directory available at this study’s start [56]). Schools designated as “alternative” or “special education only” were excluded, given the expected different contexts and needs of these settings. This resulted in a population of 51,919 public elementary schools in the US identified. Expecting a 5–10% response rate [58], 10,000 schools were randomly selected from this list using a random number generator. Chi-square tests indicated that school characteristics (state, community type, size, Title I designation) were not significantly different between the sample of invited principals (*n* = 10,000) and the population (*N* = 51,919), indicating that this invited sample could provide representative respondents. A team of undergraduate research assistants (RAs) reviewed publicly available information to identify principal email addresses for each of the 10,000 randomly selected schools. If a principal’s email address could not be identified, the school was removed and a new school was randomly chosen from the master file to take its place.

#### 2.3.2. Survey Administration

Study authors emailed survey invitations to the randomly identified potential participants in January 2023. Informed consent information was provided within the survey and the email invitation. Approximately 1500 email invitations bounced back. RAs helped identify additional randomly selected principal email addresses to replace these invitations so that 10,000 were delivered in total. At the end of the survey, participating principals had the opportunity to enter a drawing for a USD 100 gift card to either Amazon or Starbucks. Data collection was closed 1 April 2023.

### 2.4. Analyses

All quantitative analyses were run using SPSS version 28 [59]. RQ1 (what portion of schools implementing multi-tiered SMH programs report using teams to support these programs?) was analyzed using descriptive and group differences (Chi-square tests of independence [60,61]). The relevant sample (schools that use teams to implement multi-tiered SMH programs; *n* = 270) is well-powered (>0.80) to detect small and medium effect sizes using Chi-square tests [62]. RQ2 (what are common characteristics of SMH teams, including how often schools with multi-tiered SMH teams report using current best practices for SMH teaming?) was answered by looking at the percentage of schools that reported certain team characteristics (e.g., number of members on the team, external partner presence on the team) and use of each best practice.

RQ3 (what best practices, team characteristics, and school characteristics relate to SMH team member engagement?) was analyzed using linear regression. First, bi-variate relations between school/team characteristics and mean team member engagement/retention scores were assessed using analysis of variance (ANOVA) and independent sample t-tests [60,61]. School/team factors significantly associated (*p* < 0.05) with member engagement were then simultaneously entered into a linear regression model to assess their impact on member engagement while accounting for multiple possible predictors. Of note, the dependent variable consisted of the average of two 5-point Likert scale survey questions. While debate exists about the appropriateness of linear regression with ordinal dependent variables [63], it is possible for linear regression to provide an approximation of relations between predictors and ordinal dependent variables with normally distributed data and no fewer than five values [64,65]. As the mean engagement scores (comprising the average engagement and retention ratings) in this study meet these assumptions (see more below), linear regression is appropriate for this exploratory analysis [66]. The sample for these analyses (*n* = 280) is sufficient to detect a medium to large effect size with power greater than 0.80, α = 0.05, for a model with up to seven or eight predictors [62], indicating that the current sample is well-powered for this analysis.

## 3. Results

### 3.1. Responses and Missing Data

In total, 435 principals started the survey (4.35% of those invited). Eight participants provided consent but no other data and were removed from the analyses. An additional 75 participants answered only the first question (does your school implement multi-tiered SMH programs?) and provided no other data. Principals whose school did not provide multi-tiered SMH services were more likely to have quit the survey; χ^2^(1, *n* = 427) = 3.871, *p* = 0.049. This could indicate that the set of full responses under-represents schools without multi-tiered SMH programs. However, this had little impact on the findings, as the research questions focus on respondents who *do* provide these multi-tiered SMH services. Because these 75 respondents provided no additional data, they were excluded from the analyses. Missing data patterns across quantitative survey questions were assessed for the remaining 352 respondents. Table 2 summarizes the missing data analyses. Overall, missingness was low, and Little’s Missing Completely at Random (MCAR) test was not significant for either survey route (e.g., schools with/without teams), indicating that missing data do not have systematic patterns (or missingness is unrelated to the observed variables). Given the low amount of missing data, single imputation was used to address missing data in the analyses [67].

### 3.2. RQ1. Portion of Schools Using Teams

Of the 352 principals who completed the survey, 89% (*n* = 314) reported that their schools offer multi-tiered (preventive and treatment) SMH services. Most of these principals (89%, *n* = 280) use teams to organize such services. Table 3 shows the results of Chi-square tests of independence that assessed associations between team presence and school characteristics with two categories. School size was also significantly correlated with the presence of SMH team (χ^2^(2) = 8.59, *p* = 0.014), where large (*n* = 101, expected 97.2) and medium schools (*n* = 118, expected = 115) were more likely to have teams compared to small schools (*n* = 60, expected = 66.9). While SMH teams were significantly more likely among schools in urban/suburban communities, large schools, and schools with school-dedicated funds for SMH, Phi values indicated that the associations between these school characteristics and team presence are small and may account for little overall variance in team presence.

### 3.3. RQ2. SMH Teams’ Use of Best Practices

Table 4 summarizes teams’ characteristics and use of SMH teaming BPs. Participants had opportunities to write-in specific “other” recruitment or incentive strategies they use to engage team members. “Other” recruitment strategies (23 comments) included supporting staff-driven grassroots efforts, rotating members based on project focus, using contracts/memorandums of understanding (MOUs), asking staff with availability, or hiring new specialized staff. “Other” incentives (16 comments) included additional funding, professional development hours, and classroom coverage. The most commonly reported BPs included having a building administrator present and having access to school-wide data systems, while using “how-to-team” training was least-reported.

### 3.4. RQ3. Impact of School/Team Characteristics on Member Engagement

Overall, mean member engagement for the sample (*n* = 280) was 3.66 (*SD* = 0.88). Skew and kurtosis of mean engagement scores reflected a normal distribution. To assess the impact of school and team characteristics on member engagement scores, initially, independent sample t-tests were conducted to identify school and team characteristics significantly associated with member engagement. The presence of funding from the district (*t*(280) = −2.135, *p* = 0.034), the use of “other” SMH frameworks (*t*(280) = −3.237, *p* = 0.004), the number of team members (*t*(280) = −3.370, *p* < 0.001), the use of EBP guidelines (*t*(280) = −2.830, *p* = 0.005), access to resources (*t*(280) = −4.170, *p* < 0.005), interdisciplinary presence on the team (*t*(280) = −2.793, *p* = 0.006), and participation in team training (*t*(280) = −2.530, *p* = 0.012) were significantly associated with member engagement. One-way ANOVAs were used to test potential relations between school size and member engagement. School size and member engagement scores were not significantly associated, *F*(2, 276) = 0.507, *p* = 0.678. The results of non-significant t-tests between other school characteristics and member engagement can be seen in Appendix A. The full results of *t*-tests between team characteristics and member engagement can be seen in Appendix B.

A multiple linear regression model was used to assess the cumulative impact of the seven factors found to be significantly associated with mean member engagement scores. The results indicate that these seven factors significantly predicted member engagement within this sample, *F*(7, 272) = 6.529, *p* < 0.001, and account for approximately 12.2% of the variation in member engagement scores (Adjusted *R*^2^ = 0.122). Upon accounting for the variation in other predictors in the model, the number of team members (*t* = 2.538, *p* = 0.012) and access to resources (*t* = 3.556, *p* < 0.001) significantly predicted member engagement scores. Mean member engagement scores increased for teams with six or more members (compared with five or fewer members). Teams with access to resources had higher mean engagement scores than teams without access to resources. The full results of the multiple regression are shown in Table 5. The presence of funding from the district, the use of “other” SMH framework(s), the use of EBP guidelines, interdisciplinary presence on the team, and participation in team training did not significantly predict member engagement scores. The review of standardized predicted values, standardized residuals, and predictor variable inflation factors indicated that the model met assumptions for multiple linear regression.

## 4. Discussion

The results of this study provide insights into the feasibility of multi-tiered SMH teaming and BP usage and initial insight into factors that may impact team member engagement across a wide, diverse sample of elementary schools. The results present both unique findings that add to the literature base and findings consistent with prior research.

### 4.1. Feasibility of Multi-Tiered SMH Teaming and Best Practice Use

The authors found no prior studies that assessed SMH team presence among a diverse sample of elementary schools who implement multi-tiered SMH programs. Given frequent recommendations for SMH teaming in conceptual articles and frameworks, it was hypothesized that most schools who provide multi-tiered SMH programs would use teams. Within this sample, a vast majority (89%) of schools used SMH teams to organize multi-tiered SMH programs, indicating context validity of the SMH team approach. Teams were more common among schools in urban/suburban communities, schools with 300+ students, and schools that have dedicated school funding for SMH activities. As prior research has shown that rural and smaller schools often struggle with securing resources for non-academic student programs [68], it is unsurprising that this also likely impacts SMH teaming.

Five of the six best practices assessed in this study were reported by approximately two-thirds of teams (building administrator on team, access to school-wide data system, use of evidence-based guidelines, access to resources, and interdisciplinary representation), indicating context validity and consistent uptake of these recommended practices. Approximately one-third of teams represented in this sample reported participating in training relevant to SMH team tasks, which is a considerably smaller portion of schools compared with those that use the other best practices. Existing research on training for SMH team members (e.g., [28]) features training provided by third-party partners (e.g., university partners, research partners). While the current study did not directly assess the mechanisms through which training was provided, school systems (e.g., school districts, state education departments) may benefit from partnering with community practitioners to better support and train school building personnel who serve on these SMH teams. The results of the regression analysis in this study further support this need.

### 4.2. Supporting SMH Team Member Engagement

Contrary to the research team’s hypotheses, the provision of incentives or accommodations to team members was not significantly associated with team member engagement scores. Instead, mean member engagement scores were significantly associated with the presence of district funding, using “other” SMH frameworks, team size, using EBP guidelines, having access to resources, having interdisciplinary representation (three or more disciplines), and participating in team training. These associations are consistent with prior research indicating that funding and best practice use enhance overall team functioning.

When all covariates were placed in one regression model, only team size and access to resources remained significantly associated with higher mean member engagement scores. Access to resources showed the strongest association with member engagement when accounting for other variables. Given the large body of research describing the benefits of interdisciplinary teamwork, it was surprising that interdisciplinary presence did not remain significantly associated with member engagement in the regression model; instead, team size was more related to engagement. It may be that having “more hands” available to share the work allowed team members to avoid burnout and stay engaged with team activities above and beyond the presence of specific disciplines. Given the overlap of shared knowledge among disciplines commonly present on SMH teams (e.g., school counselor, school psychologist, school social worker, educators, etc.), it may be that having the personnel capital available adds a higher level of advantage than the nuanced perspectives of multidisciplinary personnel. While this does not negate the importance of interdisciplinary teamwork to support SMH programming, it may indicate that when resources are more constrained, administrators could benefit from adding members to the team to reach five or more *engaged* and *motivated* members rather than prioritizing representation from every impacted discipline in the school environment.

Alternatively, it is possible that having a larger SMH team (five or more members) is also indicative of a school having more resources or staff with availability, relevant expertise, and interest in SMH initiatives. This could account for part of the association between team size and engagement. If so, this finding continues to show the importance of building resources (e.g., staff and funding) and buy-in among potential contributors. In other words, schools should focus on building readiness before pursuing initiatives, such as implementing comprehensive SMH programs (e.g., [69,70]). It is also possible that larger teams allow for more dissemination of responsibilities, limiting the burden that team activities place on individuals and perhaps contributing to higher member engagement and retention as reported in this study. Related to the association between access to resources and member engagement, it is not surprising that when given adequate tools to complete tasks, team members are able to be more engaged. Overall, the results of this study are consistent with existing readiness and capacity-building frameworks (e.g., [71]) that emphasize the need for adequate capacity, resources, and motivation/engagement to build the foundation for successful implementation of SMH teams and, subsequently, SMH programs. These findings provide initial insight regarding factors that may impact SMH team member engagement (conceptualized as active participation and retention on the team in this study). The results indicate the following implications for research and practice and should be considered within the context of study and measure limitations, as further described.

### 4.3. Limitations

Several important limitations impact the interpretation of these results. First, the questions used to measure “member engagement” in this study may not include all aspects of member engagement. As schools are known to be under-resourced and have high demands, measure brevity was prioritized to reduce principal burden in responding. Two survey questions focused on active participation and member retention but did not include a more nuanced measure of ongoing behaviors and attitudes that could also constitute engagement. As only two items were used, the variability in engagement scores may be limited and not fully reflect nuanced differences in member engagement. While the two items used to measure engagement showed acceptable reliability and variability (meeting assumptions for multiple linear regression [64,65,66]), the findings provide an initial estimate of relationships between team/school characteristics and member engagement. Additional research is needed with a more nuanced measure (including multiple items) of member engagement to best understand these relations. Nonetheless, it is promising that this study’s results are consistent with findings about business teams that indicate that additional personnel and monetary resources [45] are associated with better team functioning.

Second, this study sought input from a nationally representative sample of elementary school principals. The response rate from invited principals was lower than expected (4.35%), and several respondents did not complete the survey or provide data relevant to the study (e.g., do not offer multi-tiered SMH programs). Therefore, most results come from a sample of principals that represent less than 1% of the full population of public elementary schools within the US. While the sample was well-powered for the analyses presented here and seems to represent a more diverse sample of schools than prior research on SMH teams, it is likely that the full breadth of SMH teaming experiences in elementary schools is not captured within these results. Specifically, medium-sized schools, rural schools, and schools from midwestern and northwestern states were overrepresented in this sample while small schools, urban schools, and schools from states in the middle south of the US were underrepresented. In addition, the current study used principal report to learn about SMH teams due to the logistical advantage of reaching principals and their frequent inclusion on SMH teams as well as their general school knowledge. However, this may bias results towards principals’ unique perspectives, particularly when principals are not actual members of the SMH teams. Future research may attempt to include perspectives of more team members to prevent this possible bias and gather more robust perspectives on teaming experiences.

### 4.4. Implications for Practice

Taken together, the main findings of this study indicate several practice implications at the school and larger community levels. Ultimately, school systems need to continue to build support systems that allow SMH teams to function efficiently and sustain successful multi-tiered SMH programs. Within schools, administrators are encouraged to consider their available funding and allocate a realistic budget specific to SMH activities (as school funding was associated with team presence). Schools are also encouraged to include at least six members on the team to help disseminate responsibilities, decrease burnout, and encourage ongoing engagement in team activities. Related to training, schools are recommended to identify partners (e.g., community mental health providers or national organizations, such as the National Center for School Mental Health) or higher-education institutions that can provide “how-to-team” training to team members, thus helping the members build individual capacity to adequately complete team projects. These partners, as well as external funding sources (e.g., state, federal, private grants), may help schools secure training and materials to support teams. Overall, access to resources showed the strongest association with member engagement. Because of this, it seems important that state education departments, districts, and school buildings work together to identify and allocate resources (e.g., funding for programs and staff, materials, staff time) that are commensurate with SMH programming goals.

### 4.5. Implications for Research

In addition to practice implications, results from this study extend the literature base and highlight areas for future research. As discussed in the limitations of the study, the field would benefit from a more nuanced assessment of SMH member engagement. Second, there seems to be widespread implementation of SMH teams. Future studies should try to include SMH teams from a variety of school contexts. This may involve more field-based research (i.e., interviews, surveys) rather than focusing on teams that have high levels of research-based support that may not be typical among most school systems. In general, it would be helpful for studies to include detailed information about schools’ settings (e.g., school size, community setting, funding resources), as these factors seem to impact teaming and could be reviewed across studies in future meta-analytic designs. This could also help researchers understand the effectiveness of SMH teaming practices in different settings.

Another finding from this study was that providing “how-to-team” training was a lesser-used best practice. Research should focus not only on the efficacy of team training systems (such as TIPS) but also on the dissemination and accessibility of such trainings. In this study, a surprising finding was that the recruitment and member accommodation strategies assessed were not associated with team member engagement. It will be important to further investigate these and other factors that may impact engagement. Qualitative interviews with SMH team members may help to initially identify factors that team members perceive to impact engagement/retention.

## 5. Conclusions

This study sought input from elementary school principals to understand the feasibility of a team approach to multi-tiered SMH implementation and use of teaming BPs. The findings indicate that many schools use a team approach and have adopted the BPs currently identified in the literature. Schools’ size and community context may be associated with the feasibility of a team strategy. Schools may need more support in accessing and providing training to SMH team members. Access to resources and team size were predictive of SMH team member engagement when controlling for funding, frameworks, interdisciplinary presence, EBP usage, and training usage. Supporting schools’ access to resources and funding may help them build the capacity and readiness needed to implement SMH teams that can adequately support impactful multi-tiered SMH programs.

## Figures and Tables

**Table 1:** SMH funding sources and frameworks among participants (*n* = 352).

	% of Participating Schools
SMH Funding Source	
District	79.5
State	54.5
School	50.9
Federal	48.9
CMH organization	25.9
University partner	2.3
Other ^1^	7.1
No funding for SMH	4.3
SMH Framework	
PBIS ^2^	73.6
TIC	36.6
SAP	11.9
WSCC	7.4
Community school	6.3
ISF	2.3
Other ^3^	7.4
No guiding framework	3.1

^1^ Principals wrote in “other” funding sources including local fundraising efforts, private grants, and student health insurance. ^2^ Framework abbreviations: Positive Behavior Intervention Supports (PBIS), Trauma-Informed Classrooms (TIC), Student Assistance Program (SAP), Whole School, Whole Community, Whole Child (WSCC), Interactive Systems Framework (ISF). ^3^ Principals wrote in “other” frameworks, most of which focus on school climate, classroom management, and social–emotional learning, Capturing Kids’ Hearts, Conscious Discipline, Educational Neuroscience, Leader in Me, PAX, Responsive Classroom, Restorative Practices, Second Step, and Zones of Regulation.

**Table 2:** Quantitative missingness across survey routes.

Survey Route	Number of Responses (*n*)	% Missingness	Little’s MCAR Test
All respondents ^1^	352	0.0–1.4	χ^2^(5) = 5.050, *p* = 0.410
Route A: *SMH = yes, teams = yes*	280	0.0–2.9	χ^2^(44) = 52.552, *p* = 0.177
Route B: *SMH = yes, teams = no*	34	0.0	NA, no missing data

^1^ The 352 respondents include the 38 respondents whose schools do not provide multi-tiered SMH services and who were not included in analyses related to the research questions.

**Table 3:** School characteristics and team presence (among multi-tiered SMH schools).

School Characteristic	School Char. = Yes # W/Teams (Expected)	School Char. = No # W/Teams (Expected)	χ^2^ (df)	*Phi*
Funding				
School	155 (146.2)	125 (133.8)	10.14 (1) **	−0.180 **
District	231 (228.3)	49 (51.7)	1.620 (1)	−0.072
State	153 (155.2)	127 (124.8)	0.622 (1)	0.045
Federal	139 (140.0)	141 (141.0)	0.132 (1)	0.020
CMH Org.	73 (77.6)	207 (202.4)	3.45 (1)	0.105
University	7 (7.1)	273 (272.9)	0.024 (1)	0.009
Other	22 (21.4)	258 (258.6)	0.17 (1)	−0.023
None	11 (11.6)	169 (268.4)	0.29 (1)	0.030
School Mental Health Framework ^1^				
PBIS	212 (208.7)	68 (71.3)	1.93 (1)	−0.079
TIC	113 (109.7)	167 (170.3)	1.52 (1)	−0.070
Title I Status	204 (208.6)	75 (70.4)	3.67 (1)	−0.108
Community Type ^2^	115 (122.1)	164 (156.9)	6.79 (1) **	0.147 **

^1^ Frameworks with fewer than 5 expected in a group (SAP, WSCC, Com School, ISF, “other”, “none”) were omitted from this table as they do not meet the assumptions for Chi-square analysis. ^2^ Rural/town is represented by the School Char = Yes column; suburban/urban is represented by the School Char = No column. ** *p* < 0.01.

**Table 4:** SMH team characteristics and best practices.

	% of Teams (*n* = 280)
Team size (# members) ^1^	
0–5	50.5
6–10	45.4
11+	3.9
External members	
External partner on team	26.4
District personnel on team	22.1
Member recruitment strategies	
Require staff with certain job types	83.9
Request volunteers	37.1
Appoint/nominate staff	37.5
Other (please specify)	8.2
Member incentives/accommodations ^2^	
Working hours protected for teaming	45.4
Written into job description	44.3
No incentives/accommodations	31.8
Other responsibilities shifted away	15.4
Receive bonus/additional funding	11.4
Other (please specify)	5.4
Best practices (BPs) ^2^	
Building administrator on team	94.3
Access to school-wide data system	78.9
Use evidence-based guidelines	70.7
Access to resources	67.9
Interdisciplinary (3+ disciplines)	66.4
Participate in “how-to-team” training	31.1

^1^ While the survey asked about three team size groups, few teams had more than ten members. For the remaining analyses, these groups were collapsed into (a) teams with 0–5 members and (b) teams with 6+ members. ^2^ Participants were invited to “select all” characteristics that apply.

**Table 5:** Multiple regression model for predicting member engagement.

Predictor Variable	*b* (*SE*)	β	*t* Value
Intercept	2.80 (0.16)	-	17.71 ***
Funding: District	0.18 (0.13)	0.08	1.33
Framework: Other	0.38 (0.20)	0.11	1.87
Team Size	0.26 (0.10)	0.15	2.53 *
BP: Use EBP Guidelines	0.21 (0.11)	0.11	1.91
BP: Access to Resources	0.38 (0.11)	0.20	3.56 ***
BP: Interdisciplinary Presence	0.17 (0.11)	0.09	1.62
BP: Team Training	0.10 (0.11)	0.05	0.932

* *p* < 0.05. *** *p* < 0.001.

## Data Availability

The data from this research can be requested by contacting the following e-mail address: wargelke@miamioh.edu.

## Appendix A

**Table A1:** Group differences in mean member engagement: school characteristics.

School Characteristic	Member Engagement for School Char. = Yes Mean (*n*, SD)	Member Engagement for School Char. = No Mean (*n*, SD)	*t* Value	*p* Value
Funding				
School	3.68 (155, 0.87)	3.63 (125, 0.89)	−0.398	0.691
District	3.71 (231, 0.86)	3.41 (49, 0.95)	−2.135	0.034
State	3.73 (153, 0.90)	3.56 (127, 0.84)	−1.60	0.110
Federal	3.67 (139, 0.89)	3.64 (141, 0.87)	−0.343	0.732
CMH Org.	3.71 (73, 0.88)	3.64 (207, 0.88)	−0.594	0.553
University	4.28 (7, 0.91)	3.64 (273, 0.87)	−1.86	0.056
Other	3.73 (22, 0.75)	3.65 (258, 0.89)	−0.409	0.683
None	3.18 (11, 0.72)	3.68 (269, 0.88)	1.834	0.068
School Mental Health Framework				
PBIS	3.63 (212, 0.91)	3.74 (68, 0.77)	1.03	0.306
TIC	3.68 (113, 0.89)	3.64 (167, 0.88)	−0.314	0.754
SAP	3.57 (36, 0.79)	3.67 (24, 0.89)	0.655	0.513
WSCC	3.78 (24, 1.00)	3.64 (256, 0.87)	−0.736	0.462
Com School	3.52 (20, 0.91)	3.67 (260, 0.88)	0.696	0.487
ISF	3.65 (273, 0.87)	3.78 (7, 1.15)	−0.390	0.697
Other	4.08 (18, 0.55)	3.63 (262, 0.89)	−3.237	0.004
None	3.60 (5, 0.89)	3.66 (275, 0.88)	0.146	0.884
Title I Status	3.78 (75, 0.88)	3.61 (204, 0.88)	−1.434	0.153
Community Type (Rural/Town vs. Suburban/Urban)	3.58 (115, 0.99)	3.71 (165, 0.80)	1.200	0.232

## Appendix B

**Table A2:** Group differences in mean engagement: team characteristics.

Team Characteristic	Member Engagement for Team Char. = Yes Mean (*n*, SD)	Member Engagement for Team Char. = No Mean (*n*, SD)	*t* Value	*p* Value
Team Size (6+ Members vs. 5 or Fewer)	3.83 (138, 0.89)	3.48 (142, 0.84)	−3.37	< 0.001
External Partner on Team	3.67 (74, 0.87)	3.65 (206, 0.89)	−0.147	0.883
District Personnel on Team	3.74 (62, 0.79)	3.63 (218, 0.90)	−0.845	0.399
**Member Recruitment**				
Required	3.68 (235, 0.85)	3.54 (45, 1.01)	−0.927	0.355
Volunteers	3.61 (104, 0.96)	3.68 (176, 0.83)	0.604	0.546
Appoint/Nominate	3.66 (105, 0.91)	3.65 (175, 0.87)	−0.074	0.941
Other	3.91 (23, 0.861)	3.63 (257, 0.88)	−1.457	0.146
**Member Accommodations**				
Protected Hours	3.69 (127, 0.077)	3.63 (153, 0.90)	−0.649	0.517
Job Description	3.58 (124, 0.84)	3.71 (156, 0.91)	1.216	0.225
Shifted Responsibilities	3.67 (43, 0.79)	3.65 (237, 0.90)	−0.139	0.890
Bonus/Funding	3.51 (32, 0.95)	3.67 (248, 0.97)	0.949	0.343
Other	3.63 (15, 0.91)	3.66 (265, 0.88)	0.108	0.914
None	3.57 (89, 0.92)	3.69 (191, 0.86)	1.073	0.284
**Best Practice**				
Admin on Team	3.68 (264, 0.87)	3.28 (16, 0.92)	−1.75	0.081
School-Wide Data System	3.69 (221, 0.87)	3.54 (59, 0.92)	−1.147	0.252
Use EBP Guidelines	3.75 (198, 0.87)	3.43 (82, 0.88)	−2.830	0.005
Access to Resources	3.80 (190, 0.84)	3.35 (90, 0.89)	−4.17	<0.001
Interdisciplinary Presence	3.76 (186, 0.85)	3.45 (94, 0.91)	−2.793	0.006
Team Training	3.85 (87, 0.88)	3.57 (193, 0.87)	−2.53	0.012
